# The prohibitin-repressive interaction with E2F1 is rapidly inhibited by androgen signalling in prostate cancer cells

**DOI:** 10.1038/oncsis.2017.32

**Published:** 2017-05-15

**Authors:** S Koushyar, G Economides, S Zaat, W Jiang, C L Bevan, D A Dart

**Affiliations:** 1 Cardiff China Medical Research Collaborative, Cardiff University School of Medicine, Cardiff, UK; 2 Androgen Signalling Laboratory, Imperial Centre for Translational and Experimental Medicine, Imperial College London, London, UK

## Abstract

Prohibitin (PHB) is a tumour suppressor molecule with pleiotropic activities across several cellular compartments including mitochondria, cell membrane and the nucleus. PHB and the steroid-activated androgen receptor (AR) have an interplay where AR downregulates PHB, and PHB represses AR. Additionally, their cellular locations and chromatin interactions are in dynamic opposition. We investigated the mechanisms of cell cycle inhibition by PHB and how this is modulated by AR in prostate cancer. Using a prostate cancer cell line overexpressing PHB, we analysed the gene expression changes associated with PHB-mediated cell cycle arrest. Over 1000 gene expression changes were found to be significant and gene ontology analysis confirmed PHB-mediated repression of genes essential for DNA replication and synthesis, for example, MCMs and TK1, via an E2F1 regulated pathway—agreeing with its G1/S cell cycle arrest activity. PHB is known to inhibit E2F1-mediated transcription, and the PHB:E2F1 interaction was seen in LNCaP nuclear extracts, which was then reduced by androgen treatment. Upon two-dimensional western blot analysis, the PHB protein itself showed androgen-mediated charge differentiation (only in AR-positive cells), indicating a potential dephosphorylation event. Kinexus phosphoprotein array analysis indicated that Src kinase was the main interacting intracellular signalling hub in androgen-treated LNCaP cells, and that Src inhibition could reduce this AR-mediated charge differentiation. PHB charge change may be associated with rapid dissociation from chromatin and E2F1, allowing the cell cycle to proceed. The AR and androgens may deactivate the repressive functions of PHB upon E2F1 leading to cell cycle progression, and indicates a role for AR in DNA replication licensing.

## Introduction

Prostate cancer is the most commonly diagnosed male cancer in the Western world.^1^ Tumour growth is initially androgen-dependent; driven by the androgen receptor (AR). Currently, the mainstays of prostate cancer treatment are androgen ablation and/or antiandrogen treatment, which block AR signalling. Hormonal therapies frequently fail and patients may relapse with ‘castrate-resistant’ prostate cancer.^2, 3, 4^ Resistance results from clonal selection of cells that circumvent androgen requirement by mechanisms including AR mutation, amplification or changes in AR cofactor (coactivator and corepressor) levels.

One such corepressor is prohibitin (PHB), previously found to be downregulated by androgen treatment.^5^ PHB has multiple roles in the cell, including (i) forming a part of a chaperone in the inner mitochondrial membrane;^6^ (ii) an attenuator of Raf-Mek signalling^7, 8^ and (iii) a repressor of various transcription factors (including E2Fs and steroid receptors). Additionally, it has tumour suppressor, antiproliferative and cell cycle regulation activities. PHB has been shown to repress E2F proteins via recruitment of the chromatin-condensing proteins HDAC1, N-CoR and BRG1/Brm.^9, 10^ PHB can also repress steroid-activated nuclear receptors, for example, AR^11^ and oestrogen receptor (ER),^12^ and conversely is capable of activating p53.^13^ PHB is a potent transcriptional corepressor of AR and ERα and associates with hormone-regulated promoters in the absence of hormone, dissociating after hormone treatment.^14^ Interestingly, PHB knockdown reduces the antiproliferative actions of oestrogen antagonists and PHB recruits BRG1-containing chromatin remodelling complex to antagonist-bound AR.^15, 16^ Additionally, PHB associates with HP1 proteins, involved in the compartmentalisation of chromatin into heterochromatin and euchromatin and may facilitate DNA structural changes required for gene activation and silencing.^14^

Previously, we showed that PHB-repressed AR activity and androgen-stimulated growth of LNCaP prostate cancer cells and that RNA interference-mediated knockdown had the opposite effects, and sensitised cells to low levels of androgens, both *in vitro* and *in vivo*.^5, 17^ This strongly implicated PHB downregulation or inactivation as a mechanism for the sensitisation of prostate cancer cells to low androgen levels/low potency ligands during apparent androgen ablation.^14^

PHB has been reported to inhibit the cell cycle.^13, 18^ Previously, we generated a stable LNCaP cell line expressing doxycycline-inducible PHB cDNA, and these cells exhibited cell cycle arrest at the G1/S boundary, with a significant reduction of cells entering the S phase.^17^ Additionally, PHB accumulated on the chromatin of hormonally starved cells. Growth stimulation with hormone or serum resulted in a rapid reduction of PHB on the chromatin, followed by a resumed cell cycle entry.

AR inhibits the expression of PHB at the mRNA level via various mechanisms involving promoter repression and the induction of AR-induced microRNAs that target PHB.^19^ The PHB protein in turn inhibits AR transcriptional activity, resulting in an interesting feedback loop. However, the androgen-induced downregulation of PHB mRNA does not account for the rapid androgen-mediated dissociation of PHB from the chromatin following androgen treatment. Such a rapid compartmental movement of a protein suggests a post-translational modification. Androgen actions are known to be mediated via both genomic and non-genomic pathways in the cell, and the androgen-mediated regulation of PHB activity may be via multiple mechanisms.

The repressive association of PHB with E2Fs and RB proteins suggests a role in the G1 arrest, and the AR has been implicated in cell cycle initiation^20^ specifically at the G1/S boundary, thus bringing together the function of these proteins. However, mechanistic details as to how androgen signalling influences PHB activity so rapidly remains relatively unknown. To assess this, we examined the interaction of PHB and E2F1 and whether it could be modulated by androgen treatment.

## Results

### PHB overexpression induces cell cycle arrest at G1/S in LNCaP cells

Previously, using a pair of doxycycline-inducible LNCaP cells, expressing PHB cDNA (or PHB-siRNA) established in Dart *et al.*,^17^ we demonstrated that doxycycline-induced PHB ectopic overexpression significantly repressed AR activity in LNCaP cells and inhibited cell and xenograft growth by cell cycle repression. PHB loss, conversely, increased androgen-stimulated cell cycle entry, growth and increased sensitivity to androgens.

**Figure 1:**
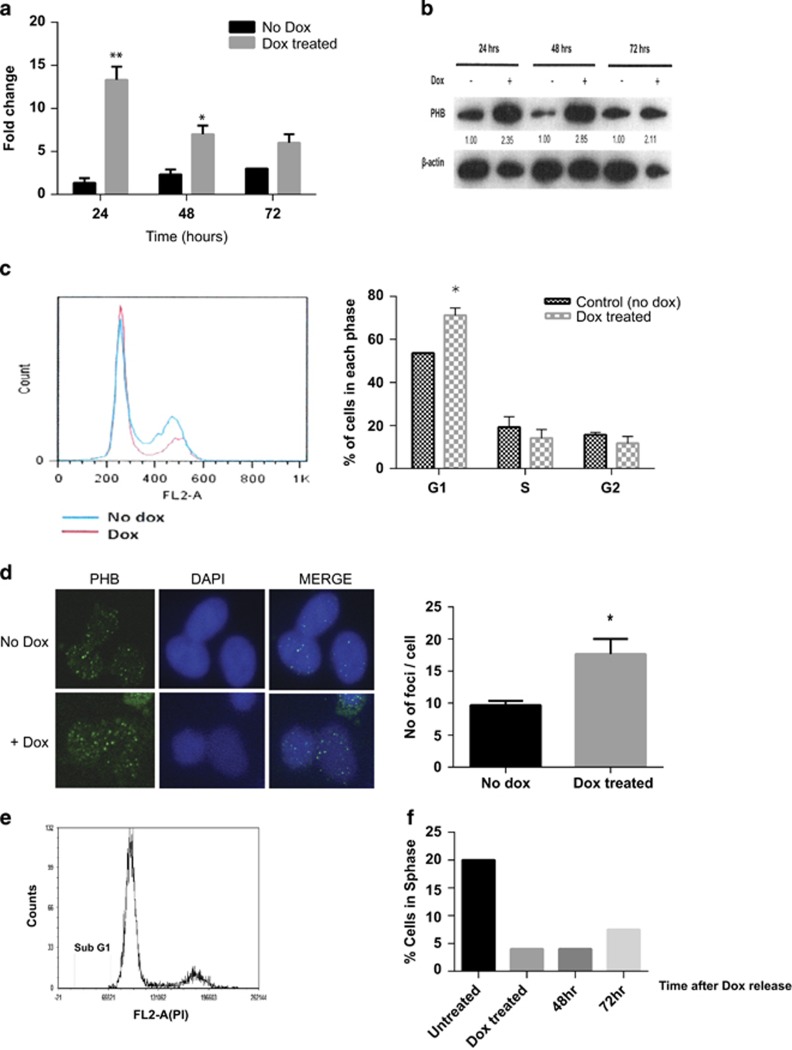
Doxycycline-induced PHB cDNA expression inhibits cell cycle entry. (**a**) Q-PCR expression analysis of PHB transcript expression from LNCaP/PHB^cDNA^ cells treated±doxycycline for 24–72 h, as normalised to β-actin, GAPDH and RPL19 housekeeping genes. (**b**) Western blot of PHB and β-actin expression in LNCaP/PHB^cDNA^ cells treated±doxycycline for 24–72 h. Densitometry data for each blot are given underneath. (**c**) FACS analysis of LNCaP/PHB^cDNA^ cells treated±doxycycline. Left-hand side represents overlaid histograms of DNA content as measured by propidium iodide fluorescence. Right-hand side shows a bar chart indicating the cell cycle distribution of LNCaP/PHB^cDNA^ cells treated±doxycycline. (**d**) Immunofluorescent staining of PHB expression and localisation in LNCaP/PHB^cDNA^ cells±doxycycline for 24 h (FITC detection), also DNA (DAPI). Associated bar chart represents number of foci per cell nucleus of LNCaP/PHB^cDNA^ cells treated±doxycycline for 24 h (from an average cell count of 100). (**e**) Cell cycle distribution of LNCaP/PHB^cDNA^ cells±doxycycline for 24 h, and washed and released and allowed to grow for 72 h. Cells show no sub-G1 apoptotic population. (**f**) Bar graph showing % of LNCaP/PHB^cDNA^ cells re-entering the S phase 72 h after doxycycline removal from the medium. All data are the mean±s.d. of three independent experiments performed in triplicate. *P<0.05 and **P<0.01 (*t*-test analysis).

Treating LNCaP/PHB^cDNA^ cells with doxycycline induced a strong ectopic expression of PHB cDNA (and resultant protein increase), within 16–24 h, which persisted for at least 72 h (Figures 1a and b). Fluorescence-activated cell sorting (FACS) analysis revealed a significant increase of cells accumulating in G1 within 24–48 h (Figure 1c). PHB overexpression resulted in an increased number of punctate chromatin-associated foci of PHB in dox-treated cells (Figure 1d). Wild-type LNCaP cells did not overexpress PHB or arrest in response to equal doses of doxycycline.

As PHB is both a nuclear and mitochondrial protein, we investigated whether PHB overexpression effects could be due to mitochondrial effects. PHB overexpression was not associated with overt mitochondrial damage or dysfunction as 3-(4,5-dimethylthiazol-2-yl)-2,5-diphenyltetrazolium bromide (MTT) and RealTime Glo assays indicated normal function per cell compared with untreated cells, normalised to cell counts either by manual counting, Cell Titre Glo or crystal violet assay (see Supplementary Figure 1). Additionally, no sub-G1 apoptotic population was detected on FACS analysis or microscopically visualised using 4',6-diamidino-2-phenylindole-stained dox-treated cells (Figure 1e). Cells arrested in G1/S by PHB re-entered the cell cycle when doxycycline was removed from the media, after 72–96 h (Figure 1f), indicating the absence of irreversible damage, or indeed permanent quiescence (G0).

### PHB overexpression represses genes involved in cell cycle progression and activates the cyclin-dependent kinase inhibitor genes

**Figure 2:**
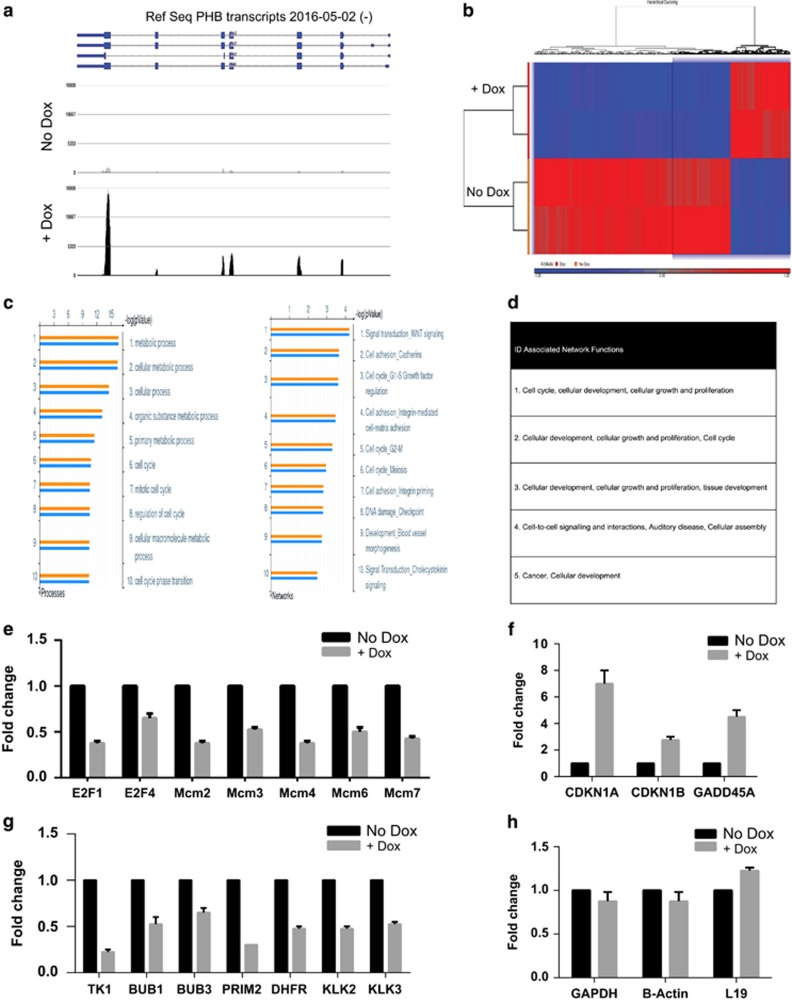
PHB overexpression downregulates genes involved in S-phase progression. (**a**) Histogram of RNA-seq reads of PHB mRNA transcript fragments from LNCaP/PHB^cDNA^ cells±doxycycline for 16 h overlayed on the gene structure of PHB (RefSeq, 2016)—indicating the doxycycline-induced overexpression of PHB RNA fragments (from integrated plasmid). (**b**) Diagram showing the hierarchical clustering analysis of the 2000+ genes either upregulated or downregulated from LNCaP/PHB^cDNA^ cells±doxycycline (∓2-fold, *P*⩽0.05). Drawn in the Partek software, blue indicates gene downregulation, and red gene upregulation. (**c**) Bar charts indicating the most significant cellular processes (left-hand side) and gene networks (right-hand side) influenced by PHB overexpression. Produced in the Metacore software from a list of genes (2000)∓2-fold, *P*⩽0.05. (**d**) Table listing the cellular networks associated with the gene expression changes in LNCaP/PHB^cDNA^ cells±doxycycline, summary from the IPA (Igenuity) and DAVID software. (**e–h**) Q-PCR validation of genes found to be involved in S-phase progression (**e**), in checkpoint activation (**f**), in DNA replication and AR responsiveness (**g**) and housekeeping genes (**h**). All data are the mean±s.d. of three independent experiments performed in triplicate. *P<0.05 and **P<0.01 (*t*-test analysis).

To determine the mechanism of PHB-induced cell cycle arrest, we used RNA-seq to identify global gene expression changes in LNCaP prostate cancer cells in response to doxycycline-induced PHB ectopic cDNA expression. To study the immediate effects of PHB overexpression, we collected cell samples at 16 h post treatment with doxycycline—this was sufficient for PHB protein overexpression but judged to avoid the cell cycle changes being secondary effects. Doxycycline-induced PHB transcripts were confirmed in the RNA-seq reads (Figure 2a) (displayed on the PHB genomic locus, however, most reads emanate from integrated plasmid located elsewhere) and resulted in significant gene expression changes. Two hundred and ninety-six genes showed upregulation (*P*⩽0.05, >2-fold) and 916 genes showed downregulation (*P*⩽0.05, <2-fold) (Figure 2b). Supplementary Excel sheet lists these genes.

### Functional categories and pathways for genes altered by PHB overexpression

The 1208 significant genes were analysed in Metacore (Thomson Reuters, London, UK), IPA (Ingenuity Pathway Analysis, Qiagen, Redwood City, CA, USA) Ingenuity and DAVID (Bioinformatics Resources 6.7; NIH, Bethesda, MD, USA) software. In Metacore, the ‘cell cycle’ regulatory Gene Ontology (GO) pathways represented 4 of the 10 top-scoring pathways, and similarly in the Network analysis (Figure 2c). IPA and DAVID algorithms also scored ‘cell cycle’ highest in four out of the five top GO pathways (Figure 2d). Other leading networks were involved in Wnt signalling and cell adhesion. Regulation of the cell cycle via E2F1–4 showed the highest network relevance in the gene set analysed (Supplementary Figures 2 and 3). Several E2F-regulated genes, for example, the *MCM* family, DNA biosynthetic precursor genes, for example, *TK1* and *DHFR*, showed strong reduction in the presence of ectopic PHB cDNA expression. Conversely, the cell cycle inhibitor genes *CDKN1A* (p21^WAF/CIP1^), *CDKN1B* (p27^KIP1^) and *GADD45A* showed upregulation.

To validate these results, we used a Human Cell Cycle PCR Low-Density Array (Qiagen), which analysed 88 genes involved in both positive and negative cell cycle regulation (Supplementary Figure 4A). This array revealed downregulation of the cell cycle/DNA replication-promoting genes—*MCM2-5*, *E2F1*, *cdc25A* and *TK1* (thymidine kinase 1) after PHB overexpression. The array also revealed upregulation of the cell cycle inhibitors p21^WAF1/CIP1^ (CDKN1A), p27^KIP1^ (CDKN1B), GADD45A and cyclin G2. These gene expression changes were then further verified to be significant by quantitative-PCR (q-PCR) of doxycycline-treated LNCaP/PHB^cDNA^ cells (Figures 2e–h). A subset of these replication regulatory genes were also analysed in the LNCaP/PHB^siRNA^ cell line, and showed opposing regulation (Supplementary Figure 4B). Further, some of these genes downregulated by PHB also showed upregulation in androgen-treated LNCaP cells (Supplementary Figure 4C)

### PHB represses MCM5 and 6 and TK1 promoter activity

**Figure 3:**
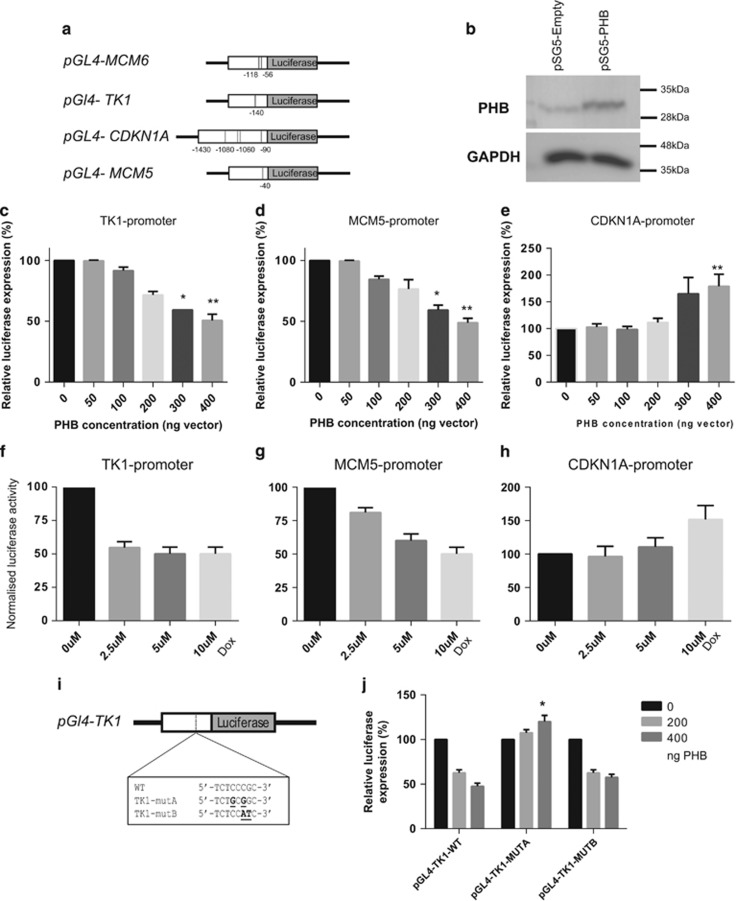
PHB represses MCM5 and 6, and TK1 promoters, while activating the CDKN1A promoter. (**a**) Schematic diagram of the promoter-reporter fusion plasmids generated linked to luciferase. The proximal gene promoter of 500–1000 bp cloned upstream of luciferase. Numbered lines indicate the position of predicted strong E2F1 binding sites (ALGGEN software). (**b**) Western blot analysis of PHB expression levels from COS-7 cells transfected with pSG5-PHB or empty vector (pSG5). (**c–e**) Luciferase activity in COS-7 cells transfected with TK1 promoter (**c**), MCM5 promotor (**d**) and CDKN1A (p21) promoter (**e**) in the presence of increasing amounts of pSG-PHB vector (0–400 ng) or empty vector control. Data were normalised to β-galactosidase activity. (**f–h**) Luciferase activity in LNCaP/PHB^cDNA^ cells treated with increasing doses of doxycycline and (**f**) TK1 promoter, (**g**) MCM5 promoter and (**h**) CDKN1A promoter. (**i**) Schematic diagram of the TK1 gene promoter indicating the E2F1 binding site and the sequences in the TK1 mutant promoters generated. (**j**) Luciferase activity in COS-7 cells transfected with TK1 and mutant TK1 promoter reporters with increasing amounts of pSG-PHB vector (0–400 ng) or empty vector. All data are the mean±s.d. of three independent experiments performed in triplicate. *P<0.05 and **P<0.01 (*t*-test analysis).

To further investigate the potential mechanism of PHB-mediated gene repression and cell cycle arrest we generated promoter-reporter fusions using 600 bp–1.5 kb fragments of the proximal gene promoters of the PHB-repressed genes *TK1* and *MCM5*, as well as the upregulated gene *CDKN1A*, were fused to a luciferase reporter (pGL4.18; Promega, Madison, WI, USA)—a schematic diagram is given in Figure 3a. The promoter-reporter fusions were transiently transfected into either COS-7 cells with increasing pSG5-PHB expression vector (or empty vector) or into LNCaP/PHB^cDNA^ cells treated with a range of doxycycline (0–10 nM), alongside a β-galactosidase control vector. The direct effect of PHB overexpression on the luciferase promoter-reporter construct was then analysed. PHB overexpression was confirmed using western blotting (Figure 3b).

PHB expression repressed TK1, and MCM5 promoter-driven luciferase activity in a dose-dependent manner, in both COS-7 (Figures 3c and d) and LNCaP/PHB^cDNA^ cells (Figures 3f and g). MCM5 protein levels were also reduced (see Supplementary Figure 5C). Additionally, PHB overexpression activated the promoter of CDKN1A, in a dose-dependent manner in both cell types (Figures 3e and h).

Analysis using the genome browser (UCSC) and *in silico* searches^21^ revealed potential E2F1 binding sites in these promoters—the location of which are seen schematically in Figure 3a. Transfection of LNCaP and COS-7 cells with a construct overexpressing E2F1 alone resulted in the upregulation of MCM5 and 6 and TK1 and repressed CDKN1A and GADD45A (Supplementary Figures 5A and B). Site-directed mutagenesis was used to change the bases in these potential E2F1 binding sites in the TK1 gene promoter. *In silico* searches showed no significant E2F1 potential sites in the resultant mutated sequences. In TK1 promoter mutant A (5′-TCTGCGGC-3′), the base changes abolished the PHB-mediated repression of this promoter, whereas with TK1 promoter mutant B (5′-TCTCCATC-3′) was still repressed by PHB (Figures 3i and j). A similar observation was seen when mutating 1 of the E2F1 binding sites in the MCM6 promoter—see Supplementary Figure 5D.

### PHB and E2F protein:protein interaction is inhibited by androgen treatment

**Figure 4:**
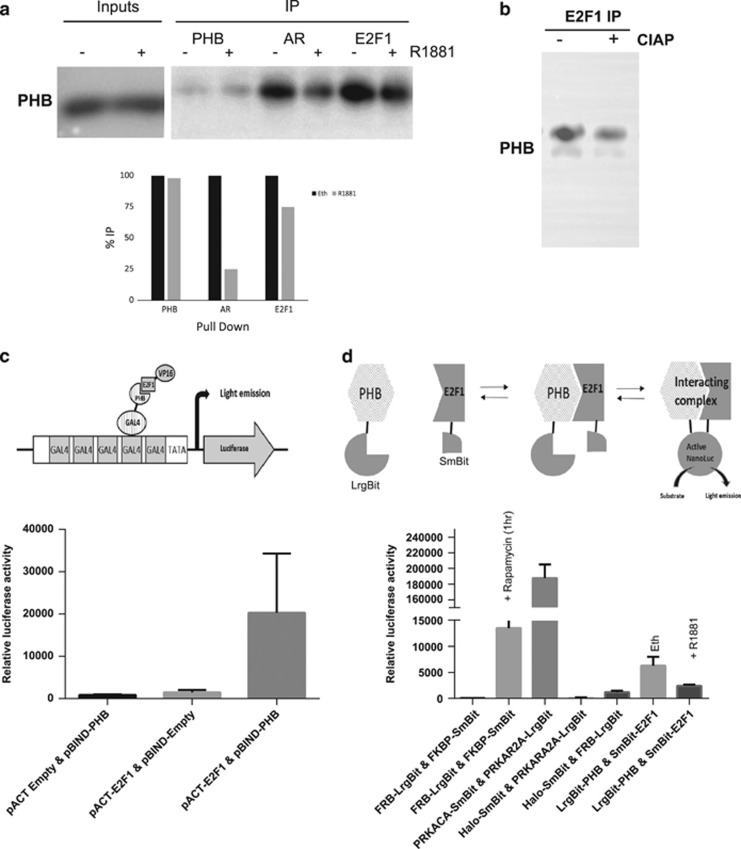
PHB:E2F1 interaction is inhibited by androgen treatment. (**a**) Western blot of PHB levels from PHB, AR and E2F1 immunoprecipitates from nuclear extracts from LNCaP cells hormonally starved for 72 h followed by treatment with 10 nM R1881 for 4 h. (**b**) Abrogation of PHB:E2F1 co-immunoprecipitation when extracts were preincubated with alkaline phosphatase enzyme. (**c**) Upper panel—schematic diagram of the Checkmate two-hybrid interaction assay for PHB and E2F1. Lower panel—luciferase activity from LNCaP cell extracts transfected with components of the checkmate assay including pBIND^Gal4^-PHB and pACT ^VP16^-E2F1, along with empty vector controls. Transfections were normalised to β-galactosidase. (**d**) Upper panel—schematic diagram of the NanoBit interaction assay for PHB and E2F1. Lower panel—nano-luciferase activity in live LNCaP cells grown in charcoal-stripped serum and transfected with components of the NanoBit assay including pPHB-LargeBit and pE2F1-SmallBit, along with empty vector controls (Halo-tagged Large+SmallBit), positive interaction controls (PRKACA:PRKAR2A) and positive rapamycin-inducible interaction control (FRB:FKBP). Cells were then treated with androgen and measured again at 2 h. All data are the mean±s.d. of three independent experiments performed in triplicate. *P<0.05 and **P<0.01 (*t*-test analysis).

PHB and E2F1 have been found to interact by various authors, and here we show they co-immunoprecipitated (co-IP) from LNCaP nuclear extracts. When IP interactions were analysed from LNCaP hormonally starved for 72 h or subsequently treated with R1881 (10 nM) for 4 h (or vehicle), the E2F1:PHB interaction was reduced by hormone treatment (Figure 4a). Also, the AR:PHB interaction was also diminished by androgen treatment. When alkaline phosphatase was introduced into the IP incubation, the E2F1:PHB interaction was also reduced (Figure 4b).

To further examine protein interaction, we used two methods: first, we used a mammalian two-hybrid assay system (Checkmate; Promega), where plasmids expressing PHB fused to yeast GAL4 DNA-binding domain (pBIND-PHB), and E2F1 fused to the herpes simplex virus VP16 activation domain (pACT-E2F1) were transfected into cells alongside a Gal4-responsive reporter plasmid (pGl5-luc) containing five GAL4 binding sites upstream of luciferase. Additionally, cells were transfected with negative control empty vector combinations and positive control plasmids expressing the MyoD-Id fusion partners. In transiently transfected LNCaP cells, luciferase activity indicated an interaction between PHB:E2F1 (Figure 4c). No signal was detected when empty vector control was used to replace the fusion plasmid.

To overcome the potentially confounding effects of such a strong repressor (PHB) and a strong activator (E2F1), a second protein:protein interaction system was used. Here, plasmids expressing PHB and E2F1 fused to either half of the two-subunit system based on the NanoLuc luciferase (NanoBit; Promega). LargeBit-PHB and SmallBit-E2F1 plasmids were transfected into both hormonally starved COS-7 cells or LNCaP cells, alongside control vectors including positive interaction control, a rapamycin-inducible positive interacting control and Halo-tagged SmallBit vectors and nonspecific/non-interaction negative control vectors. This allowed real-time analysis of the PHB-E2F1 interaction to be monitored in the presence of androgen, which was added to the LNCaP cells and light emission measured in real time.

The interaction controls showed a strong light emission as expected, and the inducible interaction control showed rapamycin-inducible interaction within 20 min—persisting for over 1 h. Hormonally starved LNCaP cells showed a modest activity indicating some direct interaction between PHB and E2F1, which was diminished by treatment with androgen after 1 h (Figure 4d).

### Androgen treatment rapidly changes the isoelectric point of PHB in LNCaP cells, but not in PC3

**Figure 5:**
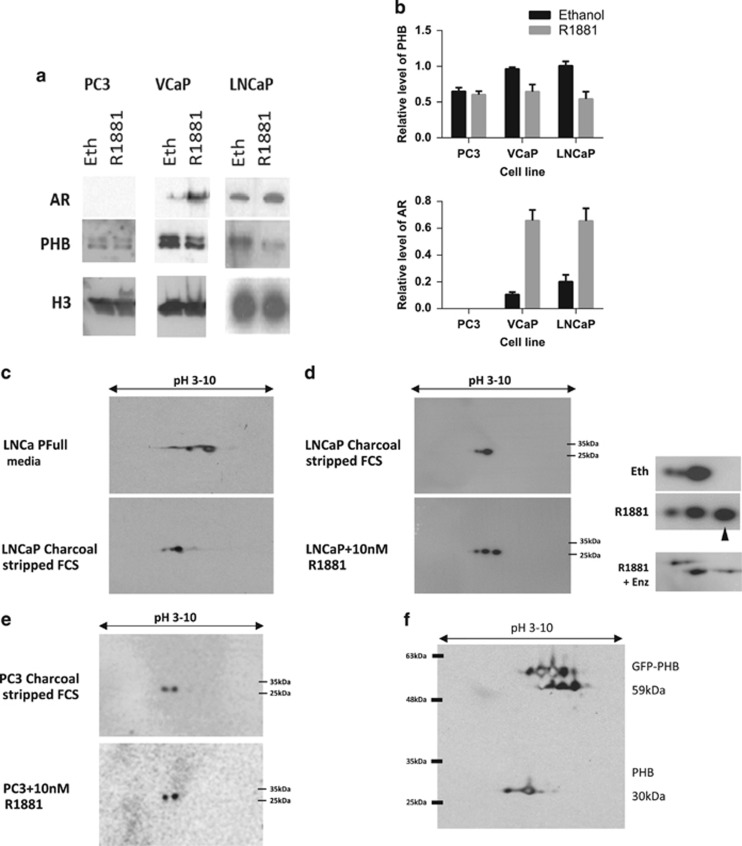
AR signalling alters PHB isoelectric charge point. (**a**) Western blot of PHB associated with the chromatin in LNCaP, VCaP and PC3 cells grown in media with charcoal-stripped serum for 72 h and then treated with ethanol or R1881 (10 nM) for 4 h. (**b**) Densitometry data for PHB (upper panel) and AR (lower) association with the chromatin in PC3, VCaP and LNCaP cells grown in media with charcoal-stripped serum for 72 h and then treated with ethanol or R1881 (10 nM) for 4 h. (**c**) 2D western blot for PHB from LNCaP cells grown in media with full serum or charcoal-stripped serum for 72 h. Blot represents isoelectric focussing of pH 3–10 horizontal and protein size vertical (4–12% gradient gel). (**d**) 2D western blot of PHB from LNCaP cells grown in media with charcoal-stripped serum for 72 h and then treated with ethanol or 10 nM R1881, or R1881 together with 10 μM enzalutamide for 4 h. Blot represents isoelectric focussing of pH 3–10 (horizontal) and protein size (vertical 4–12% gradient gel). Insert figure shows magnification and arrow indicates additional basic PHB species. (**e**) PC3 cells grown in media with charcoal-stripped serum for 72 h and then treated with ethanol or R1881 (10 nM) for 4 h. Blot represents isoelectric focussing of pH 3–10 (horizontal) and protein size (vertical 4–12% gradient gel). (**f**) 2D western blot of PHB from LNCaP cells transfected with GFP-tagged PHB, with isoelectric focussing pH 3–10 and linear 12% acrylamide gel.

The androgenic effect upon PHB translocation off chromatin in LNCaP cells was seen to be very rapid—within 1–2 h^14^ and similarly in the AR-expressing cell line VCaP (Figures 5a and b). However, in the AR-null PC3 cell line, the overall cellular level of PHB was very low and no changes were seen to the remaining chromatin-associated PHB with androgen treatment. In LNCaP cells, the downregulation of PHB by AR via promoter or miRNA pathways was too slow to account for this change (16–24 h),^5^ as was the fact that increased PHB was seen in the soluble nuclear and cytoplasmic compartments, indicating no overall change in cellular PHB levels^17^ in this timeframe.

To study this rapid and potentially non-genomic action of androgens, we hormonally starved LNCaP cells for 72 h in charcoal-stripped foetal calf serum, and then treated them with 10 nM R1881 for 4 h. These cells were then rapidly lysed either in 9 m urea/thiourea for isoelectric focussing and two-dimensional (2D) western blotting, or in array lysis buffer for protein kinase array analysis.

From LNCaP cells grown in full media, 2D western blotting for PHB showed two large spots (+2 smaller spots) (see Figure 5c, upper panel). However, from hormonally starved LNCaP cells this was seen as a single large spot with an isoelectric point of pH5 (approx), with two lesser spots (Figure 5c, lower panel and Figure 5d, upper panel). After 2–4 h of androgen treatment, the pattern of PHB changed showing two large spots with a shift towards the isoelectric point of pH 6 (approx) (see Figure 5d, lower panel). This indicated that PHB underwent a significant androgen-induced isoelectric change, but with no associated change in size. This indicates a loss of negative charge that suggests a dephosphorylation event, or a chemically neutralising modification of a previously charged amino acid, but not a large ligand/protein conjugation, for example, ubiquitin. This charge shift could also be inhibited in LNCaP cells treated with R1881 when treated with the antiandrogens enzalutamide (10 μM)—see Figure 5d. A similar charge shift pattern for PHB could also be observed in the AR-positive VCaP cells line (see Supplementary Figure 7C). The AR-deficient PC3 cell line showed two spots for PHB when grown in full, starved or starved media with added androgen. A similar pattern was also observed for the HeLa (AR-null) cell line (Supplementary Figure 7A). The spot pattern was confirmed to be PHB by repeating the 2D western blot using cells transfected with GFP-tagged PHB, in which both PHB and GFP-fused PHB could be seen. 2D western analysis also showed a shift for E2F1 protein—which underwent a rapid increased in positive charge in the same timeframe (see Supplementary Figure 7B).

### Src signalling is the major androgen-activated signalling pathway in LNCaP cells

**Figure 6:**
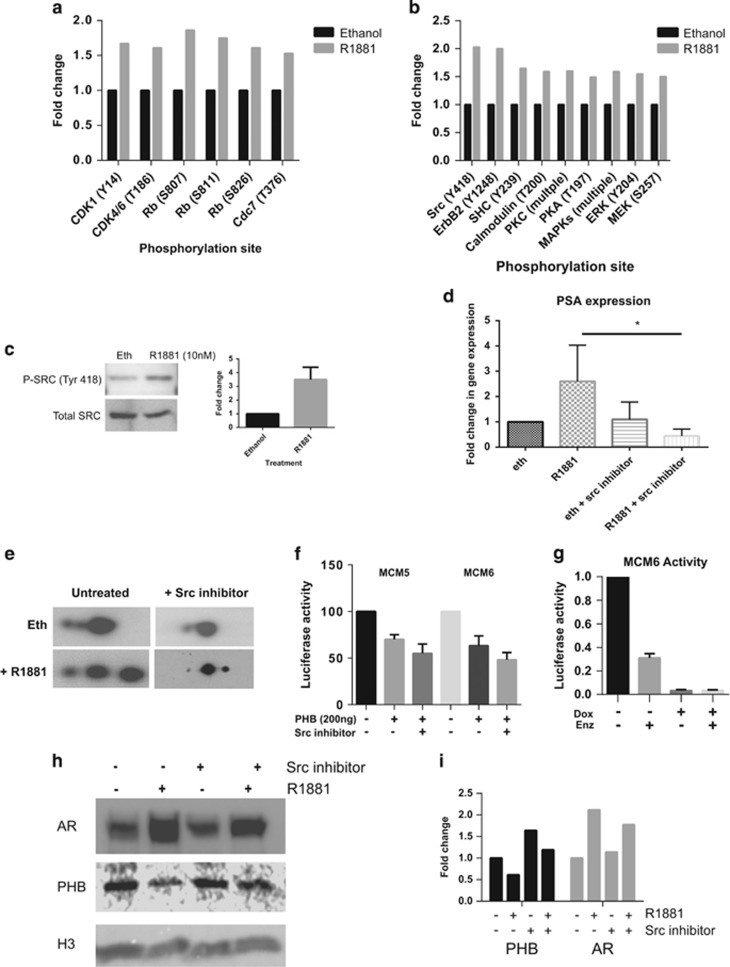
Rapid signalling cascades centred around Src kinase in androgen-treated LNCaP cells leads to PHB charge differentiation. (**a** and **b**) Kinexus protein array data from LNCaP cells grown in media with charcoal-stripped serum for 72 h and then treated with ethanol or R1881 (10 nM) for 4 h. (**a**) Phosphorylation changes on components of the cell cycle machinery at G1/S. (**b**) Phosphorylation events in intracellular cell signalling proteins. (**c**) Western blot of total Src and Src (phospho-Tyr418) from LNCaP cells grown in media with charcoal-stripped serum for 72 h and then treated with ethanol or R1881 (10 nM) for 4 h. Bar graph represents densitometry data. (**d**) Q-PCR analysis of androgen-driven PSA expression in LNCaP cells grown in media with charcoal-stripped serum for 72 h and then treated with ethanol or R1881 (10 nM) for 4 h with or without Src inhibitor. (**e**) 2D western blot of PHB from LNCaP cells grown in media with charcoal-stripped serum for 72 h and then treated with ethanol or R1881 (10 nM) for 4 h with or without Src inhibitor. Blot represents isoelectric focussing of pH 3–10 horizontal and protein size vertical (4–12% gradient gel). (**f**) Luciferase assays of COS-7 cells transfected with pSG5-PHB plasmid and MCM5 and 6 promoter reporters in the presence or absence of Src inhibitor. Transfections normalised to β-galactosidase. (**g**) Luciferase assay of LNCaP/PHB^cDNA^ cells grown in full serum, transfected with MCM6 reporter and treated with either doxycycline and enzalutamide for 24 h. Data represent the average of three independent experiments and is normalised to untreated cells. (**h**) Western blot of AR, PHB and histone H3 associated with the chromatin of LNCaP cells grown in media with charcoal-stripped serum for 72 h and then treated with ethanol or R1881 (10 nM) for 4 h, with or without Src inhibitor. (**i**) Bar graph indicating the relative densitometry of chromatin-associated AR, PHB and from LNCaP cells grown in media with charcoal-stripped serum for 72 h and then treated with ethanol or R1881 (10 nM) for 4 h, with or without Src inhibitor. All data are the mean±s.d. of three independent experiments performed in triplicate. *P<0.05 and **P<0.01 (*t*-test analysis).

To analyse potential signalling cascades that are initiated by androgen treatment, LNCaP cells were hormonally starved for 72 h and then treated with 10 nM R1881 for 4 h and rapidly lysed in strong denaturing buffer. Kinexus array (359 phosphosite-specific antibody array) analysis, on the lysates, confirmed that Src signalling pathway was the most prevalent signalling cascade in androgen-treated LNCaP cells, with several protein components having Src as the apical kinase (see Figures 6a and b). Several members of the cell cycle machinery were also phosphorylated in response to androgen. These pathways are summarised in the Supplementary Data and in Supplementary Figures 6A–C. Src was rapidly phosphorylated on Tyr^418^ by androgen treatment (Figure 6c). Tyrosine 418 is located in the catalytic domain of Src and is one of the autophosphorylation sites. Full catalytic activity of Src requires phosphorylation of Tyr^418^.^22^ The transcriptional activity of AR was also inhibited by Src inhibitor treatment (Figure 6d). Androgen treatment in the presence of Src inhibitor reduced the androgen-mediated PHB charge shift towards pH 6 (Figure 6e), and increased the repressive function of PHB in transfection assays (Figure 6f). Enzalutamide treatment of LNCaP/PHB^cDNA^ cells (in full medium) transfected with MCM6 promoter-reporter showed an increased respressive activity of the luciferase reporter (Figure 6g). Androgen treatment in the presence of Src inhibitor also reduced androgen-mediated PHB dissociation from the chromatin (Figures 6h and i).

### PHB expression levels are reduced and PHB protein is further dephosphorylated in bicalutamide-resistant LNCaP cells

**Figure 7:**
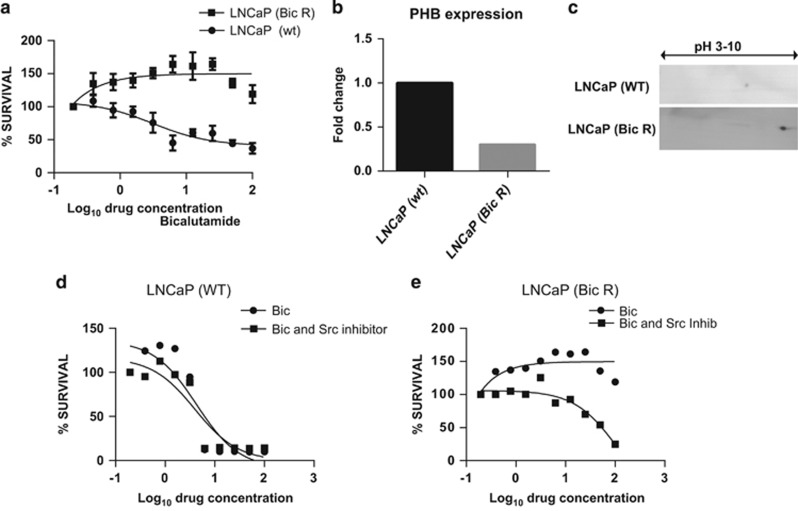
PHB expression levels are reduced and PHB protein is further dephosphorylated in bicalutamide-resistant LNCaP cells. (**a**) MTT assays on LNCaP (wt) and LNCaP cells with acquired bicalutamide resistance (Bic R) treated with increased doses of bicalutamide (μM) over 96 h. Data represent the average of three independent experiments. (**b**) Q-PCR analysis of PHB transcript levels in LNCaP Bic R cells. Levels are normalised to housekeeping genes (*RPL19*, *GAPDH* and *β-actin*). (**c**) 2D Western blot for PHB from LNCaP (wt) and LNCaP (Bic R) cells grown in media with charcoal-stripped serum for 72 h. (**d**) MTT assays of LNCaP (wt) cells with increasing doses of bicalutamide with or without Src inhibitor over 96 h. (**e**) MTT assays of LNCaP (Bic R) cells with increasing doses of bicalutamide with or without Src inhibitor over 96 h. Data represent the average of three independent experiments.

We developed a LNCaP cell variant with acquired bicalutamide resistance by growing cells in increasing bicalutamide concentrations for 6 months. The resultant LNCaP-Bic R cell line showed a proliferative response to bicalutamide (Figure 7a), whereas the wild-type LNCaP cell line is strongly inhibited. PHB has been implicated to be essential for the response to antiandrogens in prostate cancer and indeed antiestrogens in breast cancer, and PHB knockdown has been shown to reduce the efficacy of these agents. Indeed, in these bicalutamide-resistant cells we observed that PHB transcript levels were reduced by 65% (Figure 7b) and that the remaining PHB protein was charge-shifted much more towards pH 10 than in wild-type cells (Figure 7c), under hormonally starved conditions. No additional AR mutations or variants were observed in this cell line. When these cells were treated with the antiandrogen bicalutamide in the presence or absence of the Src inhibitor, we observed that the wild-type cells became slightly more sensitive to bicalutamide (Figure 7d). However, in the presence of the Src inhibitor the LNCaP-Bic R cells showed no proliferative response to bicalutamide and showed a partial response to the agent (Figure 7e).

### PHB mutagenesis identifies potential modification sites

To identify putative PHB phosphorylation sites, the complete amino-acid sequence was scanned by various software including Group-based Prediction System, Scansite, Phosphosite and Netphos2.0. These are summarised in Supplementary Table 1. This search revealed that human PHB contains 17 serine, 14 threonine and 4 tyrosine consensus sites targeted by various kinases and phosphatases.

A selection (strongest by analysis score/likelihood) of these sites in a PHB expression plasmid were mutated to alanine using site-directed mutagenesis and were used transiently transfect LNCaP cells, and *MCM5* and *TK1* endogenous genes analysed. Although not exhaustive, we did observe a reduction in PHB repressor potential with amino acids L160A, L163A and L170A (see Supplementary Figure 8). Additional loss of repression was seen to a modest degree at K83A, and at K186A, but only for the *MCM5* gene.

## Discussion

We set out to analyse how the PHB modulated the cell cycle and how its activity is regulated by AR in prostate cancer cells. PHB has been shown to recruit HP1 and HDAC proteins to the DNA on E2F1/Rb complexes, thus arresting the cell cycle at G1.^9, 14^ Using an RNA-seq-based approach, we analysed the genes repressed by PHB overexpression. Our RNA-seq and array data strongly supported that PHB inhibited the cell cycle via an E2F-related mechanism, with strong downregulation of the MCM gene family. MCMs are exclusively required for origin licensing during DNA replication.^23, 24^ Our evidence suggests that several E2F family members may be involved—as our studies indicate direct E2F1:PHB interactions, as well as GO pathways centred around E2F3/4. The E2F family has a large amount of redundancy and some E2Fs have context-dependent repressive or activating activities altering across G0>G1 >G1/S.^24^ We do not yet know if PHB interacts with other E2F family members in LNCaP cells.

Other genes involved in DNA replication and nucleotide biosynthesis, for example, *TK1* and *DHFR* were also strongly inhibited—genes with known E2F binding sites.^25, 26^ Additionally, PHB overexpression resulted in the upregulation of the checkpoint kinase inhibitors CDNK1A and B, which are normally upregulated by p53. PHB has previously been shown to enhance p53 transcriptional activity.^13^ PHB has been shown to bind the RB/E2F in a complex and to bring in repressive proteins for chromatin condensation. Mutating the E2F1 binding site in the TK and MCM6 promoters abolished the capability of PHB to repress this promoter.

E2F1 and PHB co-immunoprecipitated from hormonally starved LNCaP cell nuclear extracts. However, upon androgen treatment the resulting co-immunoprecipitation with E2F1 was reduced by 25% or more. This interaction between PHB:E2F1 was reduced in cell extracts treated with alkaline phosphatase—indicating a phosphorylation-dependent interaction. In two-hybrid assays, the interaction between PHB:E2F1 was present and could be inhibited by androgen treatment, but the interactions were not as strong as the control two proteins—indicative of PHB:E2F1 being a part of a larger complex of proteins, for example, Rb, HP1, Brg/Brm and so on, and not a simple 1:1 relationship.

We examined the effect of androgen signalling on the PHB protein itself and found that androgen treatment resulted in a rapid neutralisation of the charge upon a population of PHB protein, which may relate to dephosphorylation (although further research is required, as other events are possible). This charge shift occurred very rapidly, in a similar timescale to PHB dissociation from chromatin in androgen-treated cells. The androgen-induced charge shift could also be inhibited by the antiandrogen enzalutamide. The charge shift and chromatin dissociation was also seen in AR-positive VCaP cells. Steroids and steroid receptors have been known to elicit cellular responses in a rapid manner and are able to interact with and activate intracellular signalling molecules, for example, MAPK, ERK1/2, and raise Ca^2+^ levels^27, 28, 29, 30^—a so-called non-genomic pathway.

Kinexus array analysis for androgen-stimulated phosphorylation pathways in LNCaP cells showed several key pathways, which may be involved in this process—several with Src kinase as their main interacting hub. Although other pathways not covered by the array are equally valid, Src has been previously implicated in the non-genomic steroid receptor pathways. The AR has been shown to interact with the SH3 domain of Src.^31, 32^ Indeed, Src activity was required for full AR activity as monitored by prostate-specific antigen induction. Src inhibition also resulted in reduced androgen-mediated charge shift of the PHB protein, and dissociation from the chromatin.

In LNCaP cells with an acquired resistance to bicalutamide—a historically used antiandrogen in prostate cancer, PHB levels were reduced and the protein charge shift was seen to be more extreme. This would indicate that the dephosphorylation of PHB is involved or is a consequence of this resistance pathway. Although Src inhibition did not return the sensitivity of these cells back to that seen in the wild-type cells, it did increase the sensitivity of these cells to bicalutamide.

PHB has been shown to be downregulated in advanced prostate cancer—especially in metastatic deposits—compared with the normal surrounding cells (GEO GSE6919^33, 34^). Additionally, PHB has been implicated in the correct functioning of the antiandrogens and indeed the antiestrogens.^15^ In AR-null PC3 cells, PHB levels were very low and with the basic PHB species already present before androgen treatment. This may indicate a possible deactivation of PHB with disease progression towards androgen independence. Interestingly, online analysis of prostate adenocarcinoma DNA sequence (http://www.cbioportal.org, including TCGA analysis) described no mutations in PHB over a total of 1382 cases spread over seven clinical data sets. This may indicate that PHB inactivation by phosphorylation and gene downregulation may be more important in the mechanism of PHB involvement in prostate cancer, and may indeed be related to its essential cellular functions and highly conserved sequence across species.

Potential PHB phosphorylation site and surface accessibility analysis revealed several serine, threonine, lysine, leucine and tyrosine residues potentially targeted by kinases (and phosphatases). The repressive action of PHB was reduced upon mutation of the leucine residues to alanine—these included the nuclear export signal residues L160, L163 and L170. Additionally, but less so was the K83A (Rb binding pocket) and K186A (E2F1 binding pocket) residues. Although the mutational analysis requires much more detail, we can hypothesise that phosphorylation (or other modification) of the nuclear export signal may mask the export signal, and when removed, PHB may then come off chromatin and be exported out of the nucleus, thus allowing androgen-mediated cell cycle entry.

Analysis of the potential phosphorylation sites, listed above, via KinasePhos2.0 (http://kinasephos2.mbc.nctu.edu.tw) and Phosphonet (http://www.phosphonet.ca) implicated GRK (G-protein coupled receptor kinase) and Akt/PKB (protein kinase B) to be the most likely kinase that would target PHB for phosphorylation, but no evidence of the likely phosphatase is available. The AR/PHB interplay seems to be active on several biological levels. First, and more rapidly, pathways involving the AR and androgens rapidly dephosphorylate the PHB protein, which inhibits its repressive activity either directly or by a change in location off the chromatin. Second, the AR can downregulate the promoter activity, and third the AR can fine-tune and decrease PHB mRNA transcripts via an AR-inducible miR-27a mechanism that targets the 3'-untranslated region of PHB mRNA.^19, 35^

Interestingly, PC3 cells (AR-null) and HeLa (AR-null) cells showed both PHB species even in starved serum conditions. The fact that the AR-null PC3 cells showed no such changes indicate that PHB dephosphorylation has already occurred via an unknown mechanism, which in itself may lead cells to become independent of the AR for their cell cycle initiation. Src constitutive activation in human tumours frequently occurs and Src (Fyn and Lyn) have been demonstrated to participate in prostate tumourigenesis.^36, 37, 38, 39^ Overactive protein signalling cascades have been implicated in several modes of drug resistance including castrate-resistant prostate cancer.^40, 41^

**Figure 8:**
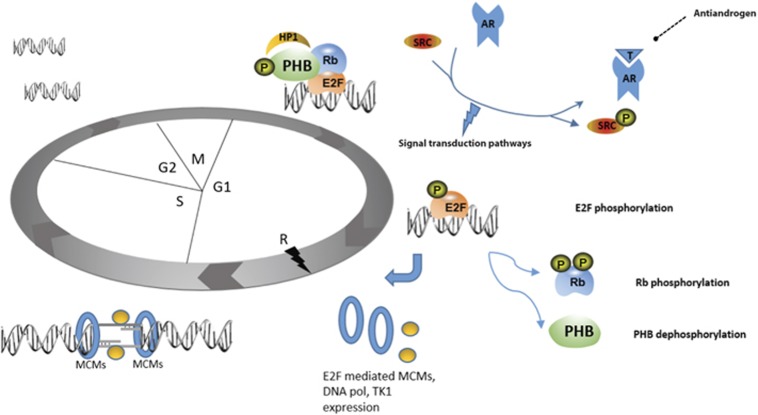
Androgen signalling inhibits PHB-mediated E2F1 repression, leading to cell cycle progression. Schematic of proposed mechanism for PHB and AR interaction at G1. Ligand binding to the AR leads to intracellular signalling cascades emanating from Src phosphorylation, leading to PHB:E2F1 disruption and dissociation of PHB from chromatin. PHB may be potentially dephosphorylated, while E2F1 and Rb are phosphorylated. E2F1 then goes onto stimulate transcription of genes required for S-phase entry, for example, MCM2-5, DNA polymerases and nucleotide biosynthesis gene, for example, *TK1*.

PHB seems to function as a scaffold, which recruits chromatin-modifying enzymes to the DNA, for example, HP1 and HDACs, thus repressing gene activity. PHB may repress E2F proteins, as well as other transcription factors, for example, AR and ER. Androgen treatment causes rapid changes in PHB protein charge and location, causing chromatin dissociation and allowing gene expression and cell cycle progression. Figure 8 shows our proposed hypothesis for the mechanism of PHB:AR interaction. This report also strengthens the evidence that AR may function as a replication licensing protein in prostate cancer cells as postulated by D’Antonio *et al.*^42^ and reviewed in Balk and Knudsen,^43^ especially given the androgen-mediated E2F1 charge shift (see Supplementary Figure 7B). The development of true androgen independence in prostate cancer may well be via oncogenic activation of the intracellular pathways that lead to replication licensing, bypassing AR.

## Materials and methods

### Cell culture

Cells were maintained at 37 °C in 5% CO_2._ LNCaPs were maintained in RPMI medium with 10% foetal bovine serum (First Link, Wolverhampton, UK) (ATCC CRL-1740). LNCaP/PHB^cDNA^ and LNCaP/PHB^siRNA^ cells^5^ were maintained in RPMI medium with 10% doxycycline-free foetal bovine serum (Clontech, Palo Alto, CA, USA), 12 μg/ml blasticidin (Sigma, Dorset, UK), 0.3 mg/ml zeocin (ThermoFisher, Waltham, MA, USA) and 500 μg/ml G418 (Sigma). COS-7 cells (ECACC 87021302), PC3 cells (ATCC CRL-1435) and VCaP (ATCC CRL-2876) were maintained in Dulbecco’s modified Eagle's medium with 10% foetal bovine serum. Media were supplemented with 2 mM l-glutamine, 100 U/ml penicillin and 100 mg/ml streptomycin (Sigma). Seventy-two hours before androgen exposure, the medium was replaced with the ‘starvation medium’ consisting of phenol red-free RPMI medium (or Dulbecco’s modified Eagle's medium), supplemented with 5% charcoal-stripped foetal bovine serum (First Link).

### Drug treatments

R1881 ((17*b*)-17-hydroxy-17-methyl-estra-4,9,11-trien-3-one) was dissolved in ethanol at a stock solution of 10 mM. Src inhibitor-1 (4-(4′-phenoxyanilino)-6,7-dimethoxyquinazoline, 6,7-dimethoxy-*N*-(4-phenoxyphenyl)-4-quinazolinamine) was obtained from Sigma and was dissolved in dimethyl sulphoxide.

### Cell cycle analysis

Cells were grown 24–72 h±doxycycline, and fixed in 70% ethanol. Cells were stained with 5 mg/ml propidium iodide and RNaseA treated (50 mg/ml). Cells were analysed with a FACS Calibur (Beckton-Dickinson, Oxford, UK), using linear scale forward and side scatter analysis, as well as DNA content. Single cells were gated and the cell cycle profiles measured from 10 000 events per sample.

### Transfections

Transient transfections of COS-7 cells were carried out in 24-well plates using the calcium phosphate precipitation method, using 500 ng of luciferase reporter, 50 ng of β-galactosidase control vector and 0–400 ng of PHB expression vector (or pSG5-empty) per well. LNCaP cells were transiently transfected using Lipofectamine 2000 (ThermoFisher), following the manufacturer's protocols.

### Luciferase assays

Cells were washed in ice-cold phosphate-buffered saline and lysed in reporter lysis buffer (Promega). Lysate was mixed with 20 μl of 0.2 mg/ml luciferin substrate (Promega) and light emission was captured using a Glomax multidetector (Promega). Transfections were normalised to β-galactosidase activity measured using the β-Gal Assay Kit (Promega).

### RNA extraction and reverse transcription–PCR

Total RNA samples were prepared using Trizol (Sigma) and converted to cDNA using the Reverse Transcription System (Promega). RNA quantity and quality was assessed by nanodrop spectrophotometer measurements and using a Bioanalyser 2100 (Agilent Technologies LDA UK Limited, Stockport, UK).

### Quantitative-PCR

Reactions were performed in triplicate on an ABI Prism 7900HT System (Applied Biosystems, Warrington, UK). Reactions consisted of 2 μl cDNA, 2 μl water, 5 μl 2 × SYBR green PCR Master Mix (Applied Biosystems) and 1 μl specific primers (0.25 pmol/μl). Primer details are given in the Supplementary Information. Parameters used were as follows: 50 °C for 2 min, 95 °C for 10 min, 40 cycles of 95 °C for 15 s and 60 °C for 1 min. Levels were normalised to glyceraldehyde 3-phosphate dehydrogenase (GAPDH) or L19.

### mRNA isolation and RNA-seq analysis

Oligo(dT)_25_ Dynabeads beads (ThermoFisher) were used to isolate polyA mRNA from total RNA and verified using a Bioanalyser 2100 (Agilent Technologies LDA UK Limited). The Ion Total RNA-Seq Kit v2 (ThermoFisher) was used to generate RNA fragment libraries, which were ligated to adapters for cDNA synthesis. cDNA was then amplified using Ion Xpress RNA-seq barcode primers and 3′ primer. The amplified cDNA was quantified with a Qubit analyser (ThermoFisher), and its profile checked for size distribution and peak concentration on Bioanalyser 2100.

### Template preparation and RNA-seq

Libraries were clonally amplified by emulsion PCR on Ion Sphere Particles using Ion PI template OT2 200 Kit v3 (ThermoFisher) on an Ion OneTouch 2 system (ThermoFisher). Template-positive Ion Sphere Particles were enriched using an Ion OneTouch ES (ThermoFisher), and were processed for sequencing using an Ion Proton 200 Sequencing Kit and were loaded onto a P1 chip and sequenced with an Ion Proton (ThermoFisher) using default parameters (single-end, forward sequencing). Base calling, adaptor trimming, barcode deconvolution and alignment was performed on Torrent Suite version 3.6 (ThermoFisher) using the STAR RNA-seq aligner plugin. The number of reads per sample was 15–20 million, with an average read depth of 500 reads per gene. The mean read length for all samples was 124 bp with 97% accuracy overall. The Partek Genomic Suite 6.6 software (Partek Incorporated, St Louis, MO, USA) was used to analyse the data. Reads per kilobase per million normalisation for RNA-seq^44^ was used using RefSeq transcript (2016-02-02) annotation followed by a one-way analysis of variance for gene differential expression. Gene targets showing a ∓2-fold change with *P-*values of <0.05 were put into DAVID, Metacore and IPA Ingenuity platforms for pathway and GO analysis.

### 2D western blot

Cells were lysed in urea/thiourea (9 m) and were then subjected to a 2D clean-up procedure (GE Healthcare Life Sciences, Amersham, UK). Isoelectric focussing of the samples was carried out using an Immobiline DryStrip IPG strip (pH 3-10; GE Healthcare) at 8000 V for 8 h. It was then equilibrated into a reducing solution for 15 min, and then replaced with 5 ml of alkylating solution (100 mM iodoacetamide in NuPAGE LDS sample buffer (ThermoFisher)). The IPG strip was run through a NuPAGE 4–12% Bis-Tris Zoom Gel (ThermoFisher) and transferred onto PVDF membranes for immunoblotting.

### Chromatin isolation and *in situ* cell fractionation

Procedures were carried out as described previously in Dart *et al.*^14^ Nuclei were isolated from this protocol for immunoprecipitation experiments—where they were lysed in IPH buffer (50 mM Tris-HCl, pH 8.0, 150 mM NaCl, 5 mM EDTA, 0.5% NP-40, 1 mM PMSF, 50 mM NaF, 2 mM Na_2_VO_3_ and protease inhibitor mix (Roche Products Limited, Welwyn Garden City, UK)) and 600μg of protein extracts were incubated with primary antibody and then with IgA magnetic beads. Immunoprecipitated proteins were washed in IPH buffer, and resuspended in sodium dodecyl sulphate sample buffer for western blotting.

### Immunoblotting

Twenty micrograms of cell extract was separated by sodium dodecyl sulphate–polyacrylamide gel electrophoresis and electroblotted onto nitrocellulose membranes. Nonspecific binding was blocked in tris-buffered saline with 0.05% Tween-20 and 5% (w/v) non-fat-dried milk. Primary antibodies were PHB mouse monoclonal (MS-261-PO; ThermoFisher) against GAPDH (SC-32233 (Santa Cruz, CA, USA); 1:5000) or PHB (SC-28259 (Santa Cruz Biotechnology, Inc., Dallas, TX, USA); 1:1000), MCM5 (SC-22780; Santa Cruz), E2F1 (SC-193; Santa Cruz). Peroxidase-labelled rabbit anti-mouse secondary antibodies (Sigma) were used at 1:2000. The membrane was then incubated in chemiluminescent substrate (GE Healthcare), and light emission detected by autoradiography.

### Kinexus kinase array

Cells were lysed in buffer (20 mM MOPS (pH 7.0), 2 mM EGTA, 5 mM EDTA, 30 mM NaF, 60 mM β-glycerophosphate, 20 mM Na_2_H_2_P_2_O_7_, 1 mM NaVO_4_, 1 mM PMSF, 3 mM benzamidine, 5 μM pepstatin A, 10 μM leupeptin, 1% Triton X-100, 1 mM dithiothreitol). Lysates were sent to the Kinexus Bioinformatics Corporation (Vancouver, BC, Canada) for analysis. The array consisted of 518 pan-specific antibodies (for protein expression) and 359 phosphosite-specific antibodies in duplicate.
