# Supplementary material for: The prohibitin-repressive interaction with E2F1 is rapidly inhibited by androgen signalling in prostate cancer cells
Source: Oncogenesis. 2017 May 15;6(5):e333–. doi: 10.1038/oncsis.2017.32 (PMC5523065; doi:10.1038/oncsis.2017.32)
Supplement: Supplementary Table 1 [file oncsis201732x1.docx]

| Amino acid position | Context | Score Prediction | Residue |
| --- | --- | --- | --- |
| 4 | MAA**K**VFES | 0.465* | K |
| 54 | VGEG**T**HFLI | 0.549 | T |
| 83 | VITGS**K**DLQ | --- | K |
| 108 | PRIF**T**SIGE | 0.868 | T |
| 109 | RIFT**S**IGED | 0.744 | S |
| 114 | IGED**Y**DERV | 0.466 | Y |
| 141 | GELI**T**QREL | 0.894 | T |
| 151 | SRQV**S**DDLT | 0.976 | S |
| 155 | SDDL**T**ERAA | 0.780 | T |
| 160 | ERAA**T**FG**L**I | 0.545 | T |
| 163 | TFG**L**ILDD | 0.562** | L |
| 170 | DDVS**L**THLTF | 0.628** | L |
| 186 | LTFG**K**EFT | --- | K |
| 202 | VVE**K**AEQQ | --- | K |

**Supplemental table 1**. Amino acid analysis of PHB protein sequence for kinase / phosphatase target sites – score given by phosphosite.org, phosphoNET, NetPhos 2.0 and NetNES online prediction tools. ** represents high predictive score for NES sequence.
