# Supplementary material for: The prohibitin-repressive interaction with E2F1 is rapidly inhibited by androgen signalling in prostate cancer cells
Source: Oncogenesis. 2017 May 15;6(5):e333–. doi: 10.1038/oncsis.2017.32 (PMC5523065; doi:10.1038/oncsis.2017.32)
Supplement: Supplementary Figure 1 [file oncsis201732x2.pdf]

Supplemental Figure 1.

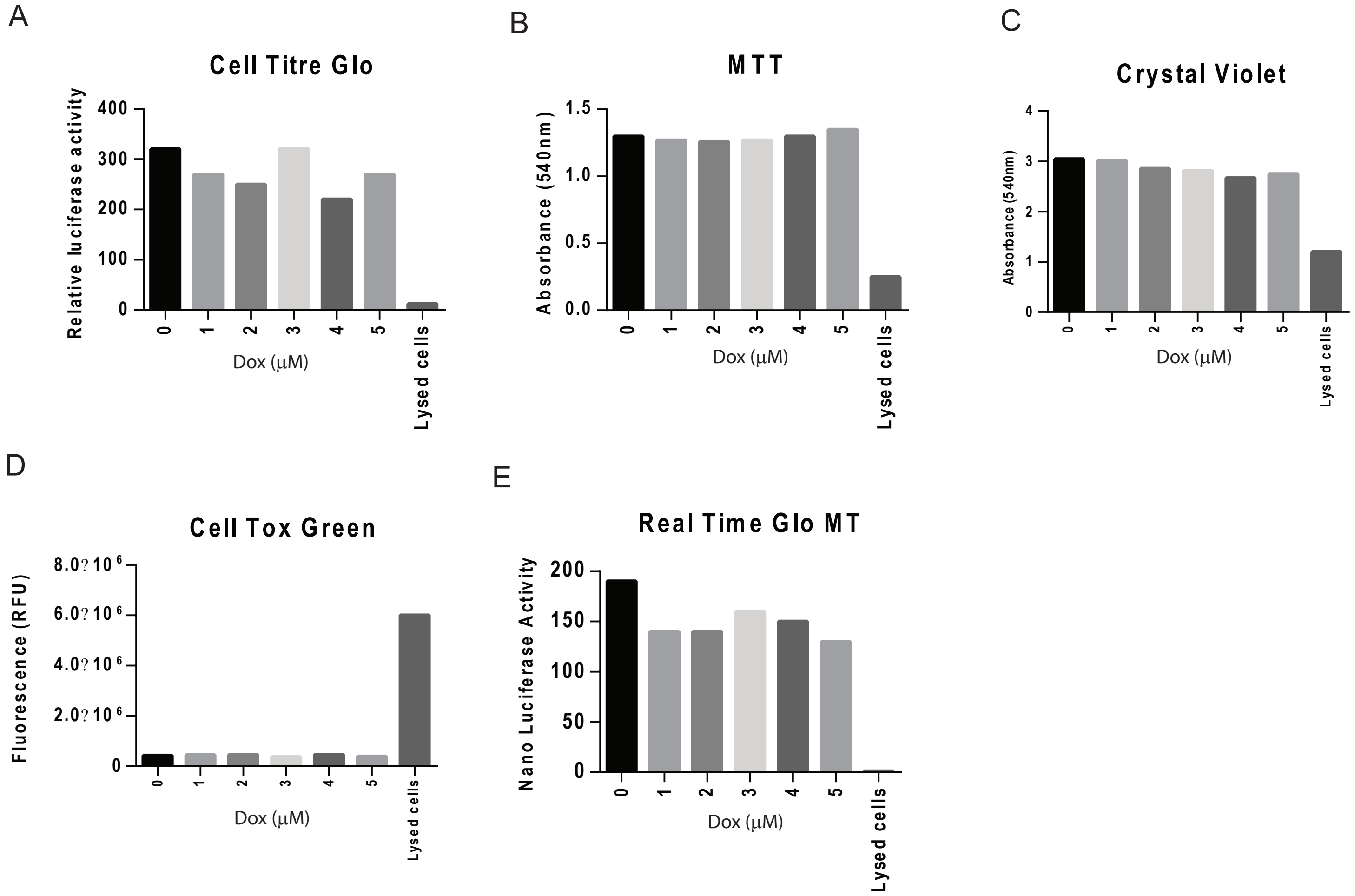


**PHB Overexpression does not induce cell death or apoptosis**.
**Cell Viability Assays**:

LNCaP/PHB cells were treated with doxycycline for 16 hours on replica plates, along with cells treated with 1% Triton X-100 as lysed control cells. Cells were then assayed as described and luminescence or fluoresence was nomalised to cell number using crystal violet staining. **A**, Cell titre Glo (Promega) - bioluminesent ATP quantification from viable cells. **B**, MTT assay for NAD(P)H in viable mitochondria [ 3-(4,5-dimethylthiazol-2-yl)-2,5-diphenyltetrazolium bromide to formazan]. **C**, Crystal violet staining for cell number. **D**, Cell Tox Green assay (Promega) to measure cell membrane permeability as a result of cell death. **E**, Real Time Glo assay (Promega) to measure viable cells which can reduce a pro-Nanoluc substrate.
