# Supplementary material for: The prohibitin-repressive interaction with E2F1 is rapidly inhibited by androgen signalling in prostate cancer cells
Source: Oncogenesis. 2017 May 15;6(5):e333–. doi: 10.1038/oncsis.2017.32 (PMC5523065; doi:10.1038/oncsis.2017.32)
Supplement: Supplementary Figure 3 [file oncsis201732x4.pdf]

Supplemental Figure 3.

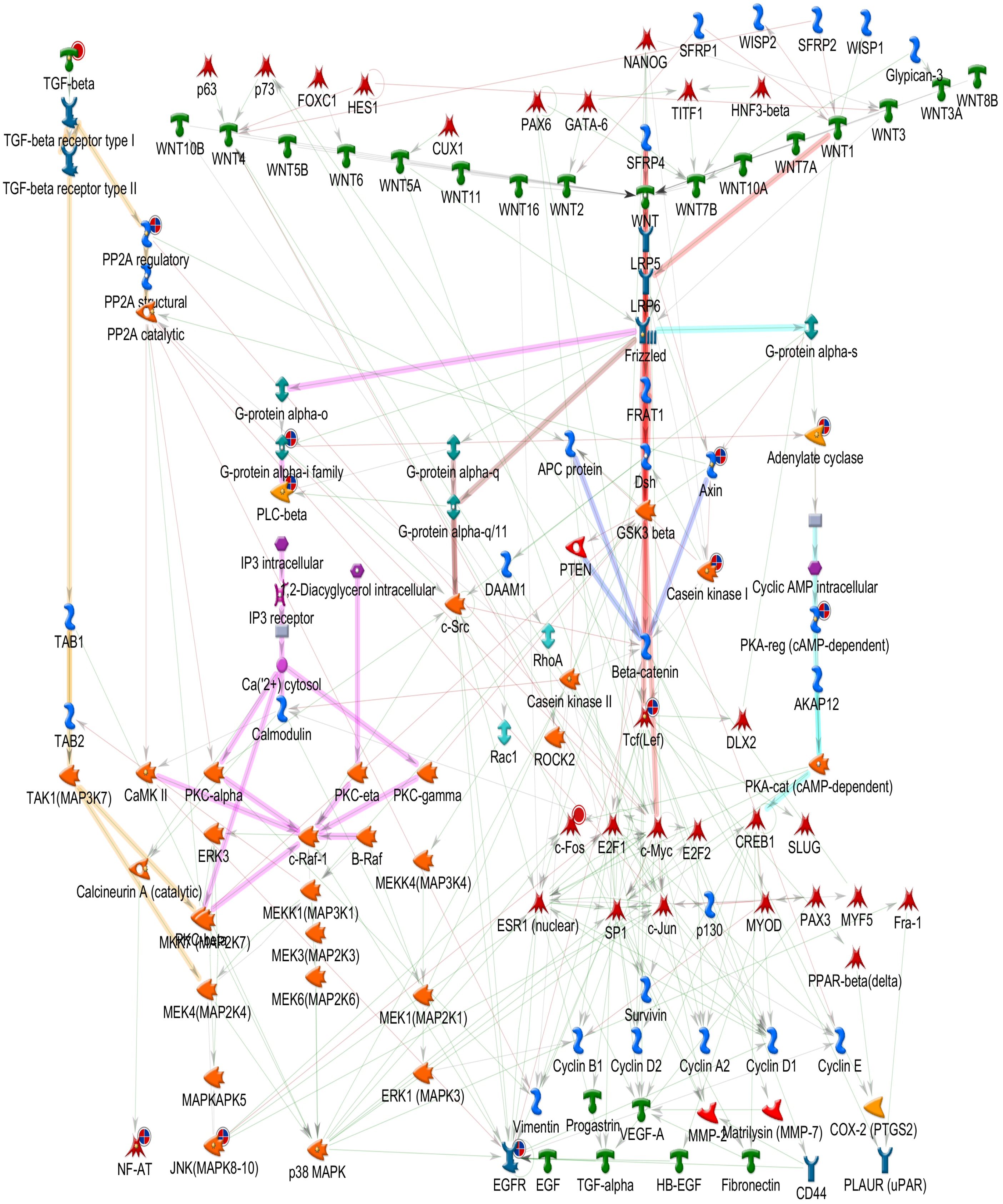

PHB Overexpression results in activation of gene involved in the Wnt > GSK3-beta, TGFB, and G protein A > Raf pathways in LNCaP cells. Network diagram created in Metacore software.
