# Supplementary material for: The prohibitin-repressive interaction with E2F1 is rapidly inhibited by androgen signalling in prostate cancer cells
Source: Oncogenesis. 2017 May 15;6(5):e333–. doi: 10.1038/oncsis.2017.32 (PMC5523065; doi:10.1038/oncsis.2017.32)
Supplement: Supplementary Figure 4 [file oncsis201732x5.pdf]

Supplemental Figure 4.

A

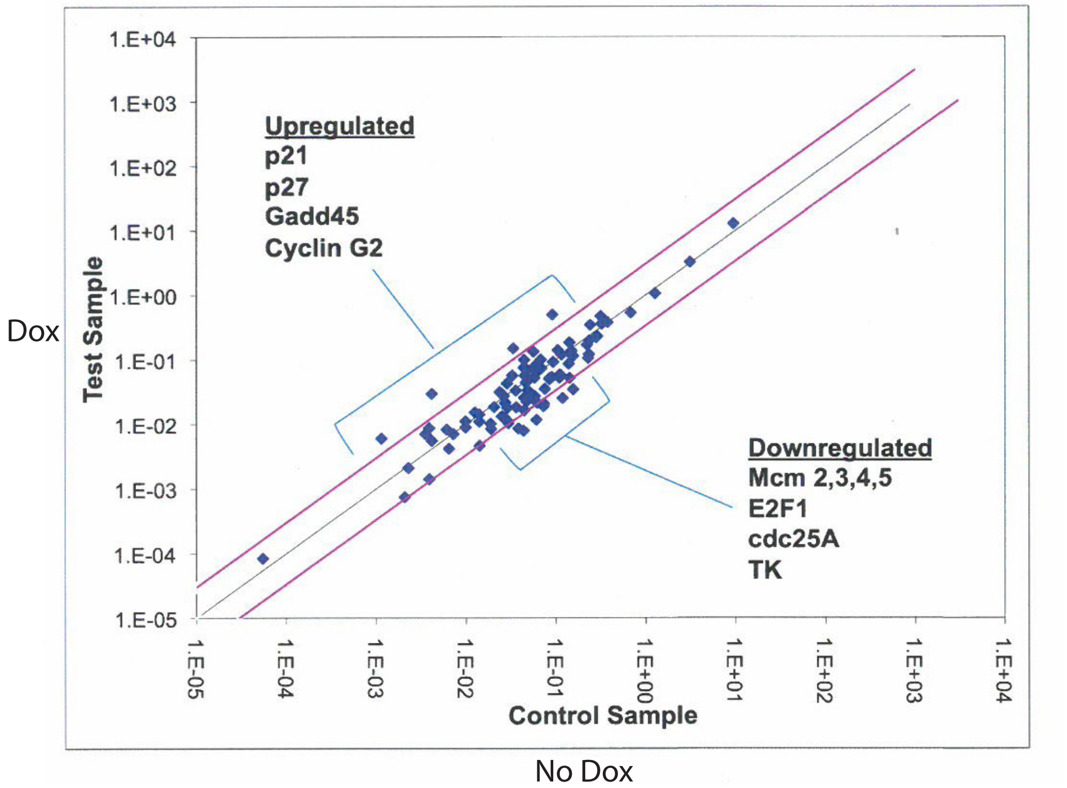

Low Density Cell Cycle Array qPCR plate (Qiagen).
Scatter diagram showing the results from the Qiagen Low density Array for 88 cell cycle regulated genes indicating relative gene of LNCaP cells treated with doxycycline (PHB overexpression) compared to untreated LNCaP cells (no dox). Red lines indicate 2 fold significance threshold. Genes with significant changes due to PHB overexpression are labelled in the figure.

B C

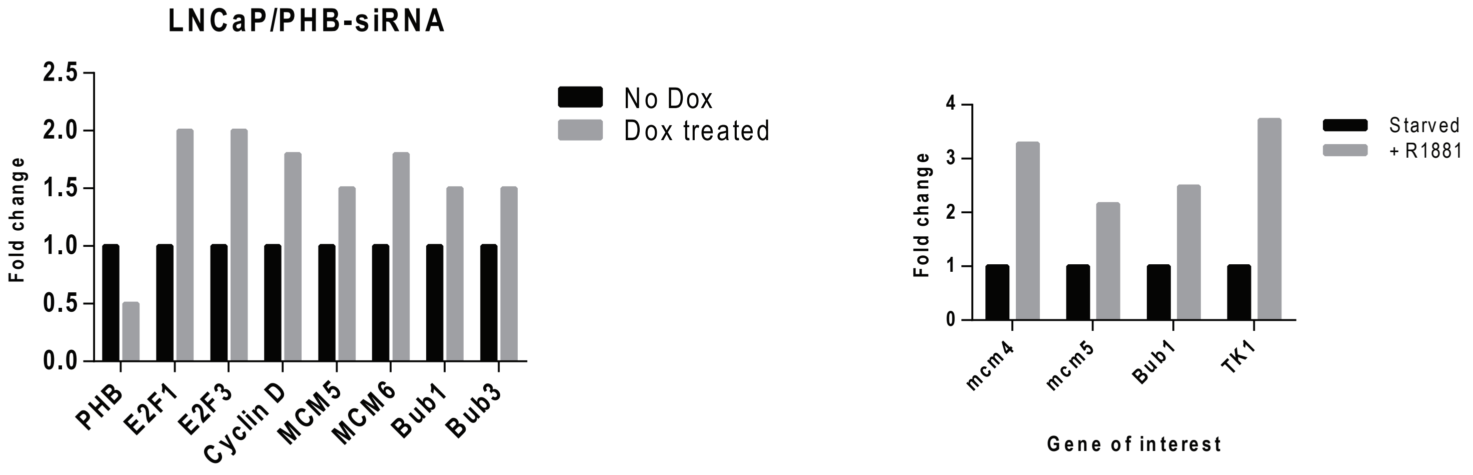

B, Q-PCR analysis of a sample of genes regulated by PHB expression. LNCaP/PHB-siRNA cells were treated with doxycycline for 48hrs. Values were normalised to Bactin, Gapdh and RPL19 housekeeping genes and then plotted relative to No-dox samples for each gene.

C, Q-PCR analysis of a sample of DNA replication genes induced by androgen-stimulated cell cycle entry. Cells were hormone-starved for 72hours and treated with R1881 for 16hours.
Values were normalised to Bactin, Gapdh and RPL19 housekeeping genes and then plotted relative to ethanol treated samples for each gene.
