# Supplementary material for: The prohibitin-repressive interaction with E2F1 is rapidly inhibited by androgen signalling in prostate cancer cells
Source: Oncogenesis. 2017 May 15;6(5):e333–. doi: 10.1038/oncsis.2017.32 (PMC5523065; doi:10.1038/oncsis.2017.32)
Supplement: Supplementary Figure 5 [file oncsis201732x6.pdf]

Supplemental Figure 5.

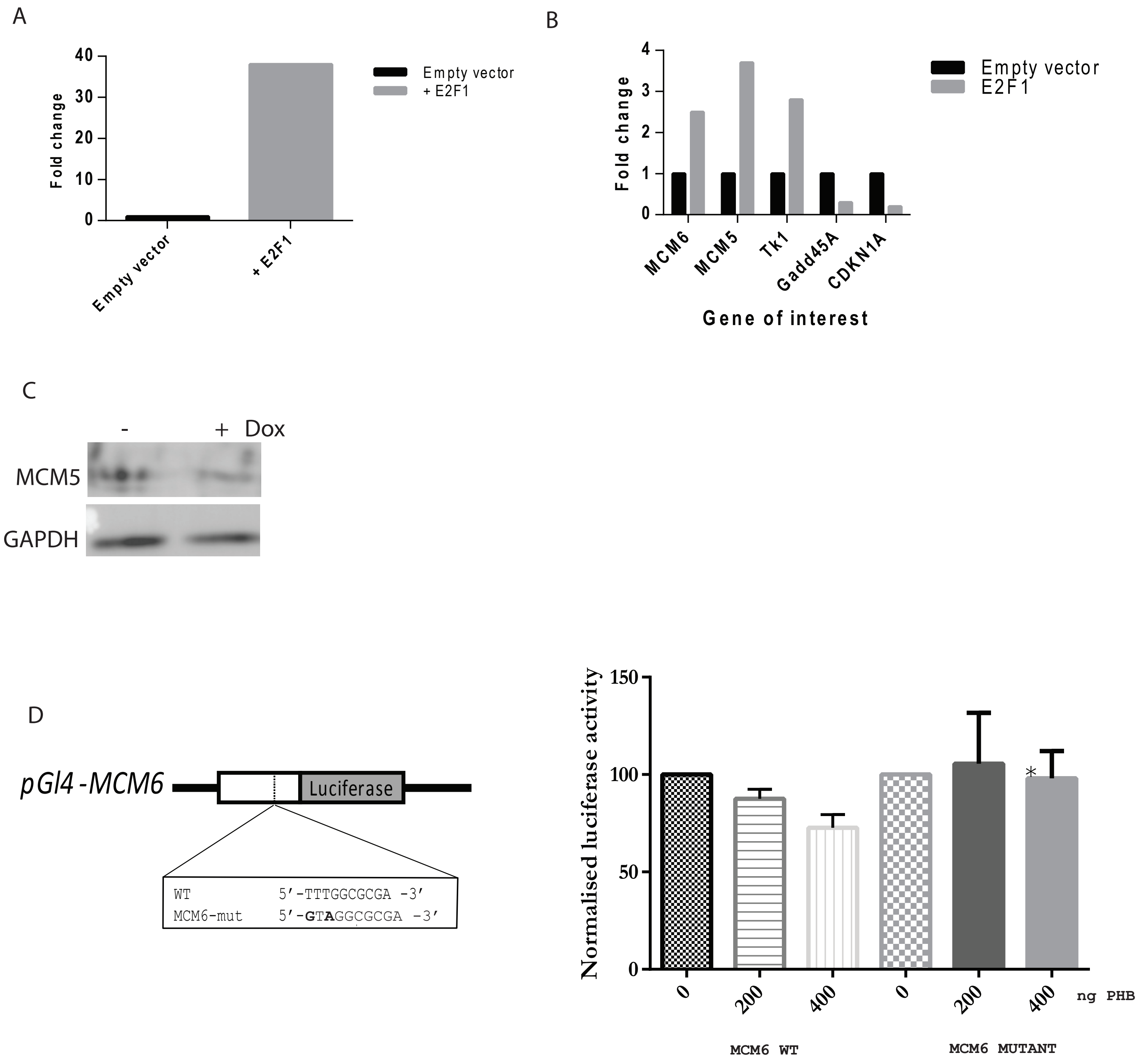

DNA replication genes MCM5&6 as well as cell cycle inhibitors Gadd45A and CDKN1A genes are regulated by E2F1.

A, Q-PCR analysis of LNCaP cells transfected with pEF6-E2F1 expression plasmid. Cells were left for gene expression for 24hours and harvested for RNA and RT-PCR for E2F1. **B**, Q-PCR analysis for a sample of DNA replication genes and cell cycle kinase inhibitor genes thought to be regulated by E2F1. Values were normalised to B-actin, Gapdh, and RPL19. **C**, Western blot of MCM5 levels from LNCaP/PHBcDNA cells treated for 24hours with or without doxycycline, compared to GAPDH loading control. D, Luciferase activity in COS-7 cells transfected with MCM6 & mutant MCM6 promoter reporters with increasing amounts of pSG-PHB vector (0-400ng) or empty vector. All data are the mean ± s.d. of three independent experiments performed in triplicate. *P<0.05, **P<0.01 (t-test analysis).
