# Supplementary material for: The prohibitin-repressive interaction with E2F1 is rapidly inhibited by androgen signalling in prostate cancer cells
Source: Oncogenesis. 2017 May 15;6(5):e333–. doi: 10.1038/oncsis.2017.32 (PMC5523065; doi:10.1038/oncsis.2017.32)
Supplement: Supplementary Figure 6 [file oncsis201732x7.pdf]

Supplemental Figure 6.

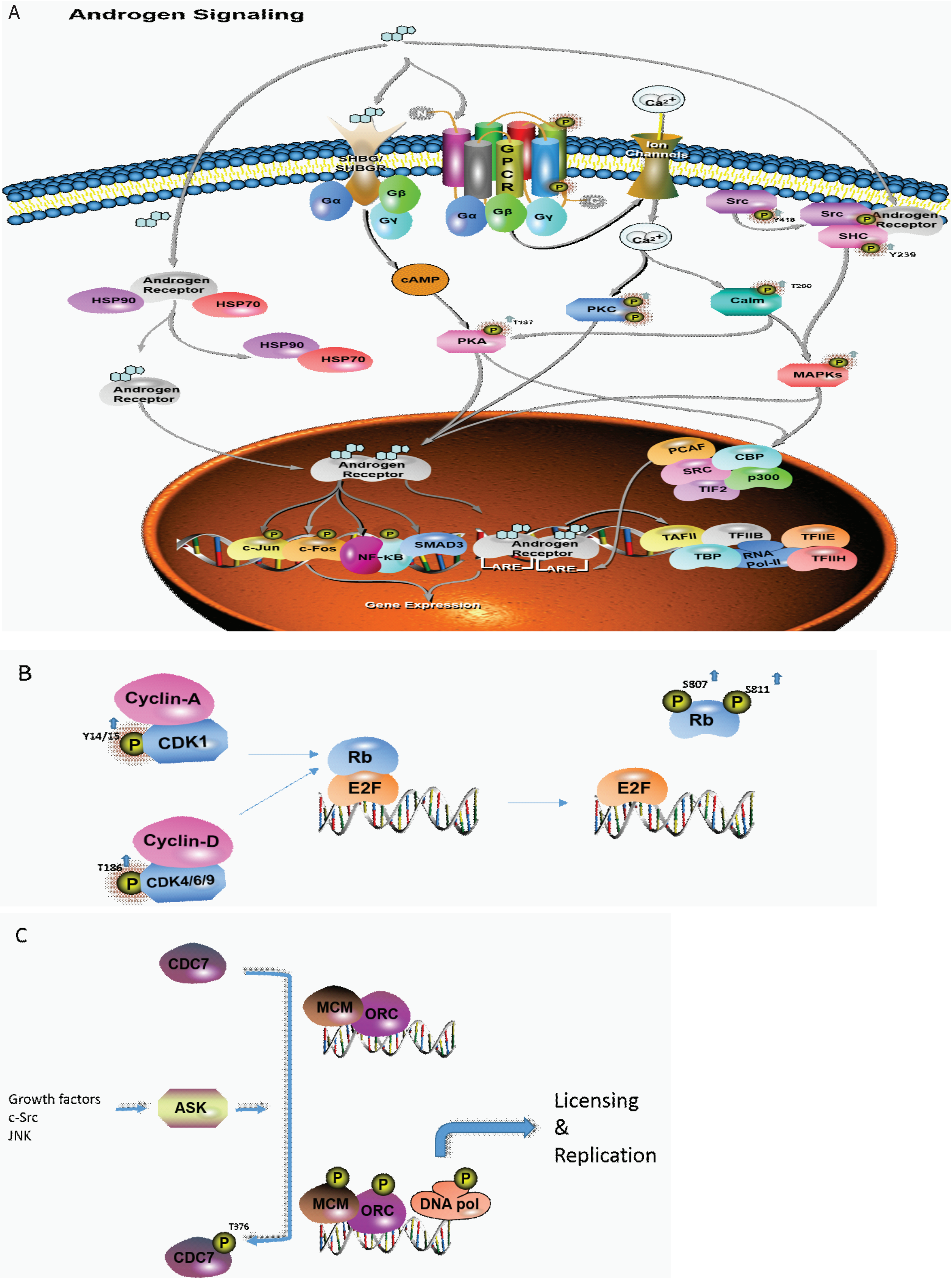

Androgen-induced phosphorylation changes in cellular pathways - results from the Kinexus data array. **A**, Cytoplasmic signalling cascade centred on Src and PKA. **B,** Nuclear phosphorylation of CDK1&4 results in phosphorylation of Rb, and **C**, phosphorylation of CDC7 promotes activation of DNA licencing proteins MCMs and ORC.
