# Supplementary material for: The prohibitin-repressive interaction with E2F1 is rapidly inhibited by androgen signalling in prostate cancer cells
Source: Oncogenesis. 2017 May 15;6(5):e333–. doi: 10.1038/oncsis.2017.32 (PMC5523065; doi:10.1038/oncsis.2017.32)
Supplement: Supplementary Figure 7 [file oncsis201732x8.pdf]

Supplemental figure 7.

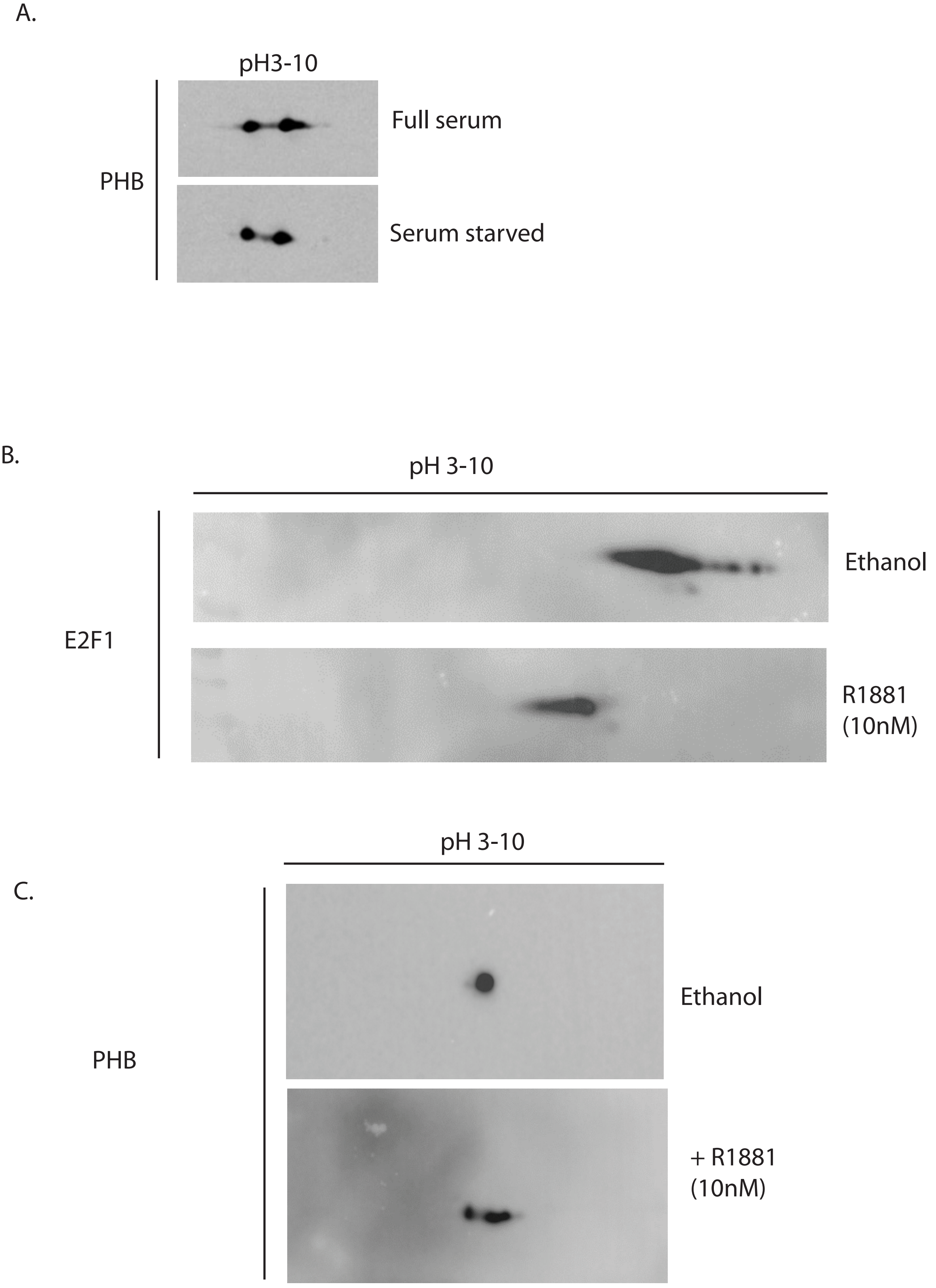

A, 2D Western blot for PHB from HeLa cells grown in media with full serum (10%) or in reduced serum (1%).
B, 2D Western blot of E2F1 from LNCaP cells hormonally starved for 72hrs followed by treatment with 10nM R1881 for 4 hours.
C, 2D Western blot for PHB from VCaP cells grown in hormonally starved medium for 72hrs followed by treatment with 10nM R1881 for 4 hours.
