# Supplementary material for: The prohibitin-repressive interaction with E2F1 is rapidly inhibited by androgen signalling in prostate cancer cells
Source: Oncogenesis. 2017 May 15;6(5):e333–. doi: 10.1038/oncsis.2017.32 (PMC5523065; doi:10.1038/oncsis.2017.32)
Supplement: Supplementary Figure 8 [file oncsis201732x9.pdf]

Supplemental figure 8.

A

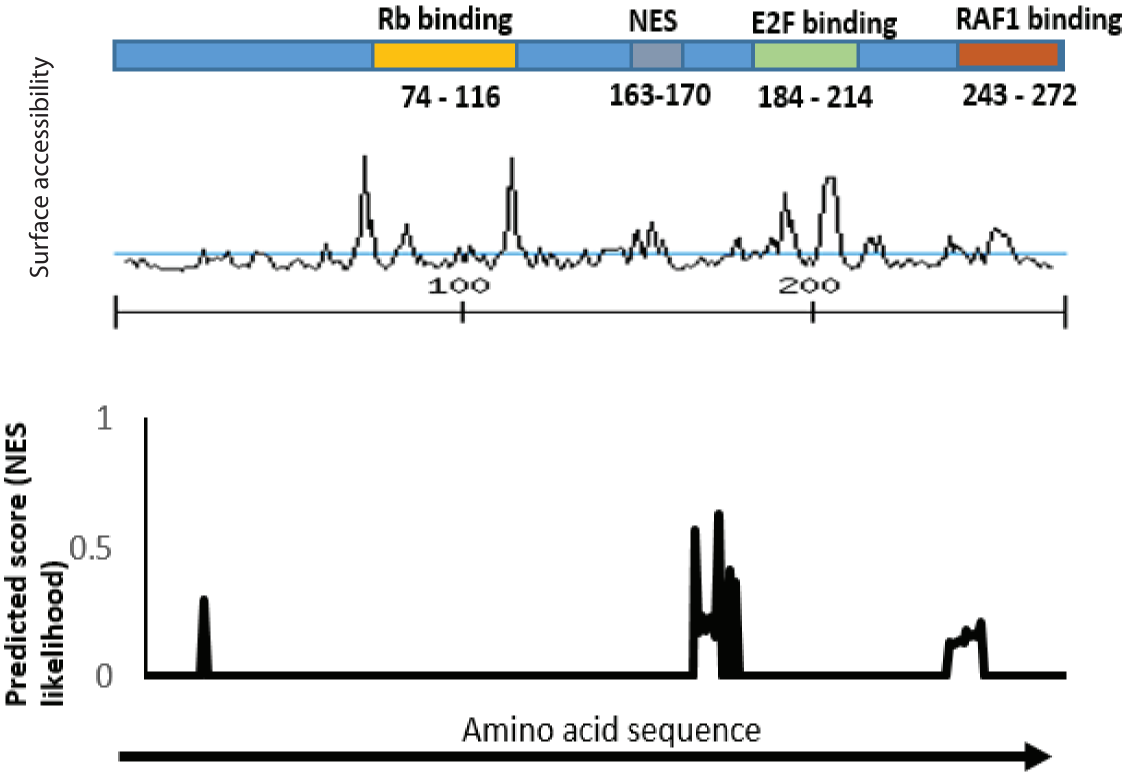

B

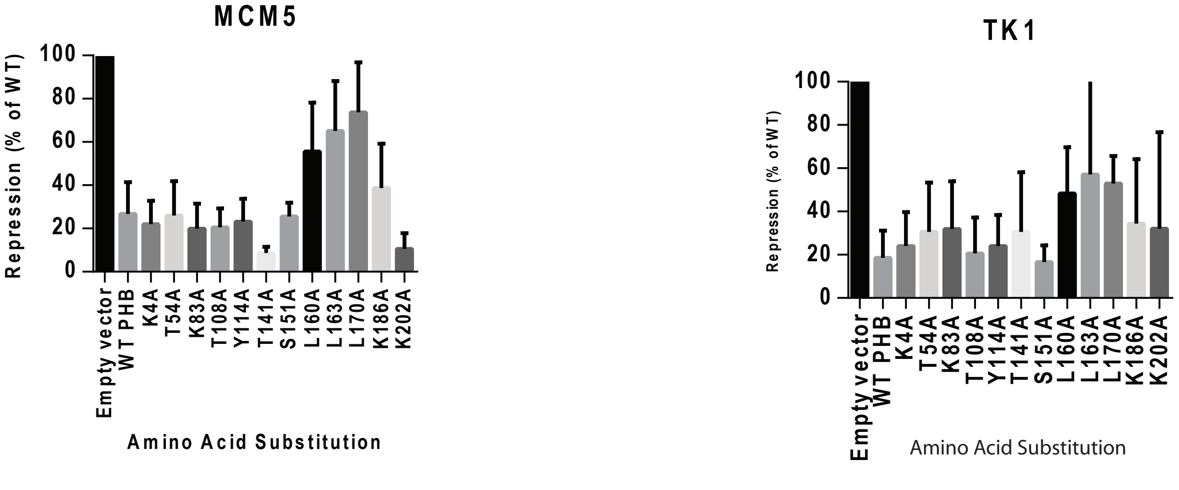

A, Schematic representation of the PHB peptide sequence with highlighted domain structure and numbered amino acids (upper panel), surface accesibility and predicted NES likelihood are given in the lower panel. Source www.phosphosite.org. **B**, Q-PCR analysis of luciferase expression from LNCaP cells transfected with pEF6-PHB plamids (WT or mutant). Data shows MCM5 and TK1 endogenous expression as compared to cellular housekeeping genes RPL19, B-actin and GAPDH. PHB expression from the plasmid was normalised to plasmid expressed blasticidin resistance gene. Repression activity of PHB phosphorylation site mutants is given as a % of WT repression.
