# Supplementary material for: The prohibitin-repressive interaction with E2F1 is rapidly inhibited by androgen signalling in prostate cancer cells
Source: Oncogenesis. 2017 May 15;6(5):e333–. doi: 10.1038/oncsis.2017.32 (PMC5523065; doi:10.1038/oncsis.2017.32)
Supplement: Supplementary Figure 2 [file oncsis201732x3.pdf]

Supplemental figure 2.

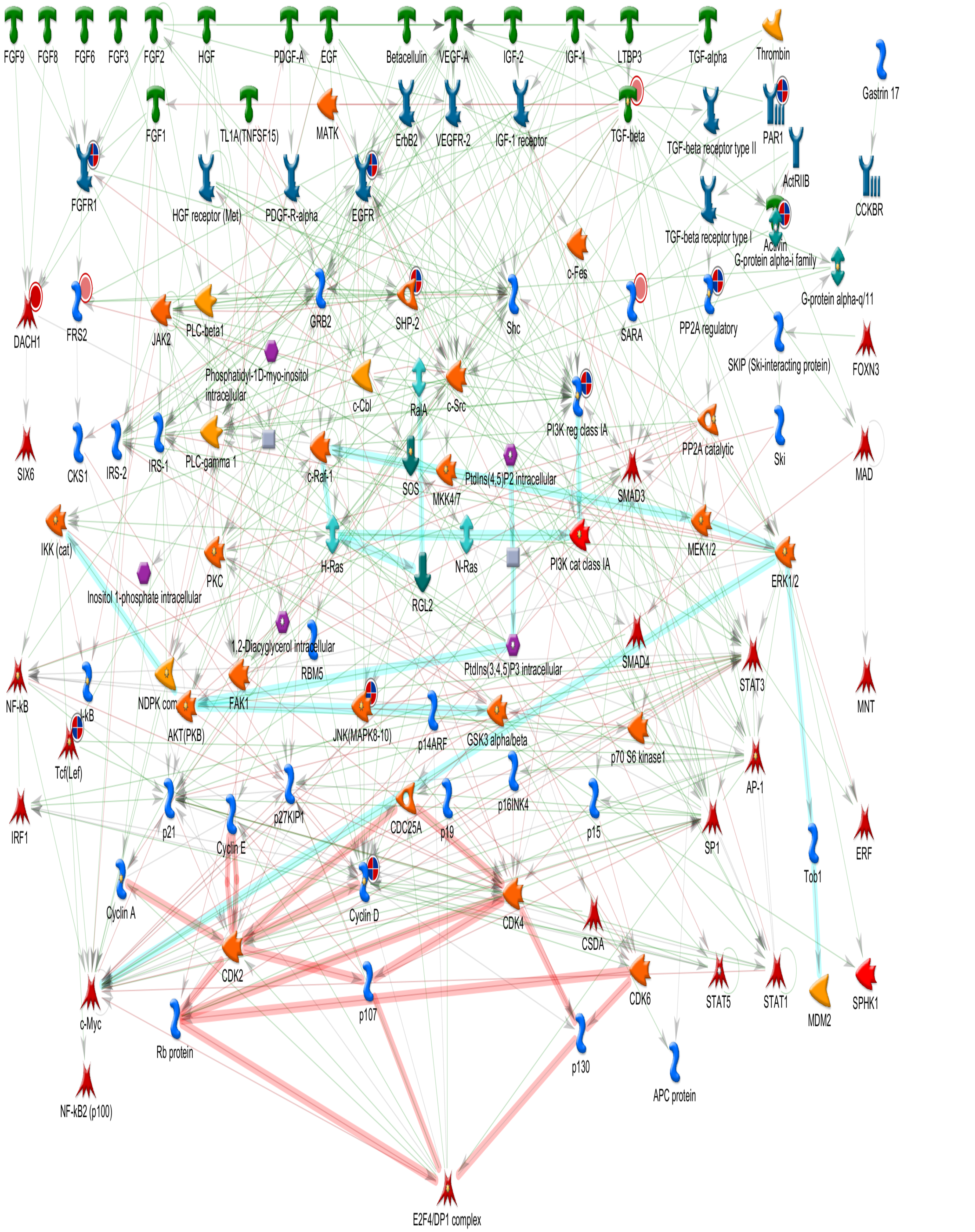

PHB overexpression results in the activation of a gene network centred upon the repression of the E2F family and the retinoblastoma (Rb, p107 & p130) family.
